# Complete Genome Sequence of Weissella cibaria Strain BM2, Isolated from Korean Kimchi

**DOI:** 10.1128/MRA.00534-20

**Published:** 2020-08-20

**Authors:** Jang-In Shin, Sang Gi Oh, Man-Seok Bang, Hee-Won Jeong, Yea-Jin Lee, Sang-Cheol Lee, Gi Soo Lee, Sungyong Kim, Han-Hwi Lee, Jae-heon Kim, Chung-Hun Oh

**Affiliations:** a Department of Oral Physiology, College of Dentistry, Dankook University, Cheonan, Choongnam, Republic of Korea; b Department of Microbiology, Graduate School, Dankook University, Cheonan, Choongnam, Republic of Korea; c Department of Medical Laser, Graduate School, Dankook University, Cheonan, Choongnam, Republic of Korea; d Clinical Trial Institute, Dankook University, Cheonan, Choongnam, Republic of Korea; e Department of Oral Physiology, Graduate School of Dentistry, Dankook University, Cheonan, Choongnam, Republic of Korea; Portland State University

## Abstract

Weissella cibaria appears to have broad-spectrum health benefits. Here, we report the genome sequence of Weissella cibaria strain BM2, which was isolated from homemade kimchi; it consists of one circular chromosome of 2,462,443 bp and one plasmid of 11,067 bp. A total of 2,337 coding sequences were predicted, including 2,117 protein-coding sequences and a G+C content of 45.06%.

## ANNOUNCEMENT

Weissella cibaria is a Gram-positive, non-spore-forming, nonmotile, hetero-lactic acid-fermenting, and catalase-negative bacillus that cannot produce dextran from sucrose (1). Members of *Weissella* are lactic acid bacteria and have been isolated from various sources, including fermented foods, Spanish sausages, Greek salami, human gut, and insect gut (2–5). Recently, certain members of the *Weissella* genus, such as W. cibaria, W. koreensis, and W. confusa, were reported to dominate during the early stage of kimchi fermentation (6, 7). In particular, W. cibaria has been reported to have probiotic properties (6, 8, 9). Here, we report the complete genome sequence of W. cibaria strain BM2, isolated from homemade kimchi.

We isolated W. cibaria BM2 from homemade kimchi. To isolate W. cibaria BM2, the soup of kimchi was diluted 1,000-fold with buffered peptone water, and then 100 μl of diluted sample was spread onto MRS agar (Difco, USA) and incubated at 30°C for 48 h under aerobic conditions. Finally, morphologically distinct single colonies were purified by repeated streak culturing on the same medium. The genomic DNA of W. cibaria BM2 was extracted using the Wizard genomic DNA purification kit (Promega, USA), following the protocol recommended by the manufacturer. The quantity and quality of isolated DNA were determined using a NanoDrop spectrophotometer. The isolate was characterized as W. cibaria based on PCR amplification of the 16S rRNA gene using primers 27F (5′-AGAGTTTGATCCTGGCTCAG-3′) and 1492R (5′-GGTTACCTTGTTACGACTT-3′), as described previously (10). A BLAST search of the sequence obtained was conducted using NCBI BLASTn. The W. cibaria BM2 gene showed 100% identity with the W. cibaria 16S rRNA gene.

To sequence the whole genome of W. cibaria BM2, a SMRTbell library with a 20-kb insert size was constructed with the PacBio DNA template preparation kit v1.0. The genome was sequenced by Macrogen (Seoul, Republic of Korea) with the PacBio RS II sequencing platform (Pacific Biosciences, USA) using a single-molecule real-time (SMRT) cell 8Pac v3 and DNA polymerase binding kit P6 reagents (11). In total, 127,616 PacBio subreads (average subread length, 10,522 bp; subread *N*_50_, 15,092 bp) of W. cibaria BM2 were generated. PacBio reads were assembled using the RS Hierarchical Genome Assembly Process (HGAP) protocol v3.0. The process consists of preassembly, *de novo* assembly with the Celera Assembler, alignment with Basic Local Alignment with Successive Refinement (BLASR) v1, and assembly polishing with Quiver v1 in SMRT Portal v2.3 (12–14). Default parameters were used except where otherwise noted. When the contig ends overlapped, contigs were connected to form circular DNA. The result of the assembly was two contigs, including one closed circular chromosome of 2,462,443 bp (G+C content, 45.1%; coverage, 380× [GenBank accession number CP027427]) and a plasmid of 11,067 bp (G+C content, 37.7%; coverage, 36× [GenBank accession number CP027428]). The genomes were annotated with Prokka v1.12b software (15), which identified 2,436 genes, 2,319 coding DNA sequences (CDSs), 28 rRNAs, 88 tRNAs, and 1 transfer-messenger RNA on the chromosome and 8 genes and 8 CDSs on the plasmid.

### Data availability.

The complete genome sequence of W. cibaria strain BM2 was deposited in GenBank under the accession numbers CP027427 and CP027428. The associated BioProject, BioSample, and SRA accession numbers are PRJNA436626, SAMN08628864, and SRR11558354, respectively.
